# Host-Species Transferrin Receptor 1 Orthologs Are Cellular Receptors for Nonpathogenic New World Clade B Arenaviruses

**DOI:** 10.1371/journal.ppat.1000358

**Published:** 2009-04-03

**Authors:** Jonathan Abraham, Jo Ann Kwong, César G. Albariño, Jiajie G. Lu, Sheli R. Radoshitzky, Jorge Salazar-Bravo, Michael Farzan, Christina F. Spiropoulou, Hyeryun Choe

**Affiliations:** 1 Department of Medicine, Children's Hospital, Harvard Medical School, Boston, Massachusetts, United States of America; 2 Laboratory of Molecular Medicine, Children's Hospital, Harvard Medical School, Boston, Massachusetts, United States of America; 3 Howard Hughes Medical Institute, Harvard Medical School, Boston, Massachusetts, United States of America; 4 Special Pathogens Branch, Centers for Disease Control and Prevention, Atlanta, Georgia, United States of America; 5 Department of Microbiology and Molecular Genetics, Harvard Medical School, New England Primate Center, Southborough, Massachusetts, United States of America; 6 Department of Biological Sciences, Texas Tech University, Lubbock, Texas, United States of America; University of California Irvine, United States of America

## Abstract

The ability of a New World (NW) clade B arenavirus to enter cells using human transferrin receptor 1 (TfR1) strictly correlates with its ability to cause hemorrhagic fever. Amapari (AMAV) and Tacaribe (TCRV), two nonpathogenic NW clade B arenaviruses that do not use human TfR1, are closely related to the NW arenaviruses that cause hemorrhagic fevers. Here we show that pseudotyped viruses bearing the surface glycoprotein (GP) of AMAV or TCRV can infect cells using the TfR1 orthologs of several mammalian species, including those of their respective natural hosts, the small rodent *Neacomys spinosus* and the fruit bat *Artibeus jamaicensis*. Mutation of one residue in human TfR1 makes it a functional receptor for TCRV, and mutation of four residues makes it a functional receptor for AMAV. Our data support an *in vivo* role for TfR1 in the replication of most, if not all, NW clade B arenaviruses, and suggest that with modest changes in their GPs the nonpathogenic arenaviruses could use human TfR1 and emerge as human pathogens.

## Author Summary

Several arenaviruses found in the New World cause hemorrhagic fever when they are transmitted from their natural reservoirs to humans. These pathogenic arenaviruses use human transferrin receptor 1 (TfR1), a protein involved in cellular iron uptake, to infect human cells. The nonpathogenic New World arenaviruses characterized thus far do not use human TfR1. We show here that two of these nonpathogenic viruses, Amapari and Tacaribe, can use animal orthologs of TfR1 to infect cells. We observe that recombinant viruses coated with the entry proteins of Amapari and Tacaribe viruses use the TfR1 orthologs of their natural reservoirs. Modest alteration of human TfR1 converts it to an efficient receptor for Amapari and Tacaribe viruses. Our findings provide insight into the potential of Amapari and Tacaribe viruses to adapt to use human TfR1 and perhaps emerge as human pathogens.

## Introduction

**Figure 1:**
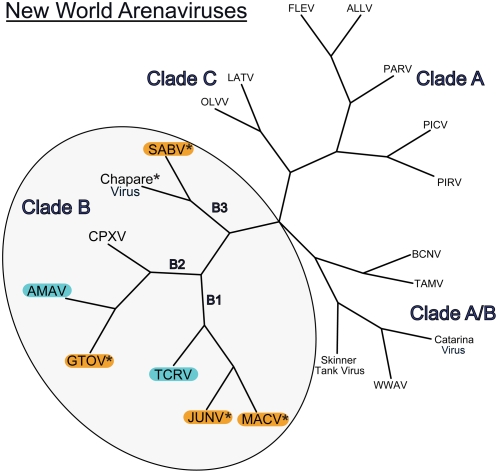
Phylogeny of New World arenaviruses based on GP sequences. All known clade B viruses are included, whereas only representative examples of other clades are shown. The sequences used in the analysis are those of full-length GPs: ALLV, Allpahuayo virus; AMAV, Amapari virus; BCNV, Bear Canyon virus; Catarina virus; Chapare virus; CPXV, Cupixi virus; FLEV, Flexal virus; GTOV, Guanarito virus; JUNV, Junín virus; LATV, Latino virus; MACV, Machupo virus; OLVV, Oliveros virus; PARV, Paraná virus; PICV, Pichindé virus; PIRV, Pirital virus; SABV, Sabiá virus; Skinner Tank virus; TCRV, Tacaribe virus; TAMV, Tamiami virus; WWAV, Whitewater Arroyo virus. The GenBank accession numbers of these viruses are indicated in the Materials and Methods section. Viruses that cause hemorrhagic fever in humans, which are only found in clade B (encircled), are labeled with an asterisk. Viruses known to use human TfR1 are boxed in orange, and the nonpathogenic arenaviruses investigated in this study are boxed in cyan [39],[40].

Arenaviruses are enveloped viruses that carry single-stranded, bi-segmented, RNA genomes [1]. The family *Arenaviridae* comprises a single genus (Arenaviru*s*) that contains at least 25 members [2]–[4]. Arenaviruses have been classified into two antigenically and geographically distinct groups, the Lassa-lymphocytic choriomeningitis serocomplex (“Old World arenaviruses”) and the Tacaribe serocomplex (“New World arenaviruses”) [5]–[7]. The New World (NW) arenavirus cluster is subdivided into clades A, B, and C. In addition, several North American viruses are recombination products of clades A and B (A/B) (Figure 1). All NW arenaviruses that cause hemorrhagic fever in humans are in the B clade. These include the Junín (JUNV), Machupo (MACV), Guanarito (GTOV), and Sabiá (SABV) viruses, which respectively cause Argentine, Bolivian, Venezuelan, and Brazilian hemorrhagic fevers with high (10–30%) case fatality rates [2],[8],[9]. A novel NW clade B arenavirus, Chapare virus, was recently isolated from a fatal case of hemorrhagic fever in Bolivia [3].

In addition to these pathogens, there are clade B viruses that do not cause disease in humans. These include the Amapari (AMAV), Cupixi (CPXV), and Tacaribe (TCRV) viruses, although TCRV may have caused a febrile illness with mild central nervous system symptoms in a laboratory worker [10]. Pathogenic and nonpathogenic NW arenaviruses do not cluster independently on phylogenetic analysis; TCRV is closely related to MACV and JUNV, while AMAV shares highest sequence similarity with GTOV (Figure 1).

The zoonotic reservoir of a given NW arenavirus has a unique geographical distribution, and each arenavirus prefers its own host [11]. Most of the natural hosts for NW arenaviruses belong to the subfamily Sigmodontinae in the family Cricetidae, which contains NW mice and rats [6],[12]. The host animals for MACV, JUNV and GTOV are *Calomys callosus* (large vesper mouse), *Calomys musculinus* (drylands vesper mouse), and *Zygodontomys brevicauda* (short-tailed cane mouse), respectively [13]–[16]. The natural reservoirs of SABV and Chapare virus have not been identified. AMAV was isolated from *Neacomys spinosus* (bristly mouse) and *Neacomys guianea* (Guiana bristly mouse), and TCRV is suggested to have a non-rodent host, *Artibeus* species fruit bats in Trinidad and Tobago [12], [17]–[19].

The arenavirus envelope glycoprotein (GP), the sole protein found on the surface of virions, is processed into three associated subunits: the stable signal peptide (SSP), GP1, and GP2 [20],[21]. SSP is a unique component of the arenaviral fusion machinery and plays a role in the transport, maturation, and pH dependent membrane fusion activity of the GP complex [22]–[25]. The GP1 subunit engages a cellular receptor(s), and GP2 mediates pH-dependent membrane fusion after viral particles are internalized into acidified endosomes [21], [26]–[29].

Two cell surface molecules have been implicated as cellular receptors for arenaviruses. Old World arenaviruses and NW clade C arenaviruses Oliveros and Latino use α-dystroglycan as an obligate receptor, while the pathogenic NW clade B arenaviruses MACV, JUNV, GTOV and SABV use human transferrin receptor 1 (TfR1) to infect cells [30]–[33]. The ability of NW clade B arenaviruses to cause disease in humans correlates with the utilization of human TfR1, a molecule that has several properties favorable to arenaviral replication and viral hemorrhagic fevers; it is rapidly endocytosed into acidic compartments, expressed on endothelial cells, and upregulated on rapidly dividing cells including activated lymphocytes [34]–[38]. We have previously shown that recombinant retroviral particles pseudotyped with the GPs of MACV, JUNV, or GTOV efficiently use the TfR1 ortholog of the rodent hosts of these viruses [39].

A number of studies have suggested that AMAV and TCRV do not use human TfR1 [40]–[43]. Blocking human cells with inactivated AMAV significantly reduces the infection of these cells with AMAV pseudovirus, but does not interfere with the infection of MACV, JUNV or GTOV pseudoviruses [43]. It remains to be shown, however, if these nonpathogenic NW arenaviruses could use the TfR1 orthologs of their principal host animals. Here, we confirm that AMAV and TCRV do not use human TfR1, and show that they nonetheless efficiently use the TfR1 orthologs of their respective animal hosts, *N. spinosus* and *A. jamaicensis*. We also show that mutation of one residue converts human TfR1 into an efficient receptor for TCRV, and replacement of four residues with those found in *N. spinosus* TfR1 converts human TfR1 into an efficient receptor for AMAV. These data show that TfR1 has an important role in the replication of nonpathogenic NW arenaviruses, and suggest that subtle changes in the GPs of TCRV and AMAV might adapt them to use human TfR1.

## Results

### AMAV and TCRV pseudoviruses do not use human TfR1

**Figure 2:**
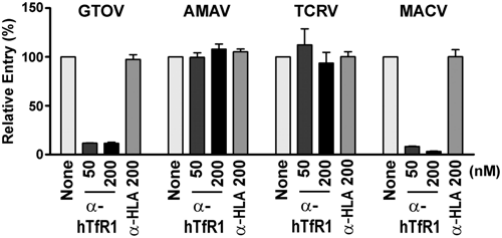
AMAV and TCRV pseudovirus entry does not depend on human TfR1. HEK293T cells were infected with GTOV, AMAV, TCRV, or MACV pseudoviruses expressing GFP in the presence or absence of the indicated concentrations of an α-human TfR1 (BD Pharmingen) or a control (α-HLA) antibody (BD Pharmingen). Infection levels were assessed 48 hr later by measuring GFP expression by flow cytometry. Mean fluorescence values were normalized to that of cells infected in the absence of antibody. GFP mean fluorescence values for virus entry in the absence of antibody were 144.0, 89.7, 92.3, and 115.0 for GTOV, AMAV, TCRV, and MACV, respectively.

We confirmed that human TfR1 is not involved in the entry of AMAV and TCRV into human cells by examining whether an α-human TfR1 antibody could inhibit the infection of HEK293T cells mediated by the GPs of AMAV and TCRV. We generated recombinant Moloney murine leukemia virus (MLV) expressing green-fluorescent protein (GFP), pseudotyped with the GPs of AMAV, TCRV, MACV and GTOV. Cells were exposed to these recombinant viruses in the presence or absence of a control antibody (α-HLA), or an α-human TfR1 antibody previously shown to inhibit infection of MACV and JUNV pseudoviruses [32]. Infection levels were assayed forty-eight hours later by flow cytometry (Figure 2). The α-human TfR1 antibody inhibited infection by GTOV and MACV pseudoviruses, but had no effect on the entry of AMAV and TCRV pseudoviruses. The entry of all four pseudoviruses was unaffected by the α-HLA antibody. These results are in agreement with previous studies showing that human TfR1 is not involved in the entry of the nonpathogenic NW clade B arenaviruses into human cells [40].

### Animal orthologs of TfR1 support entry of AMAV and TCRV pseudoviruses

**Figure 3:**
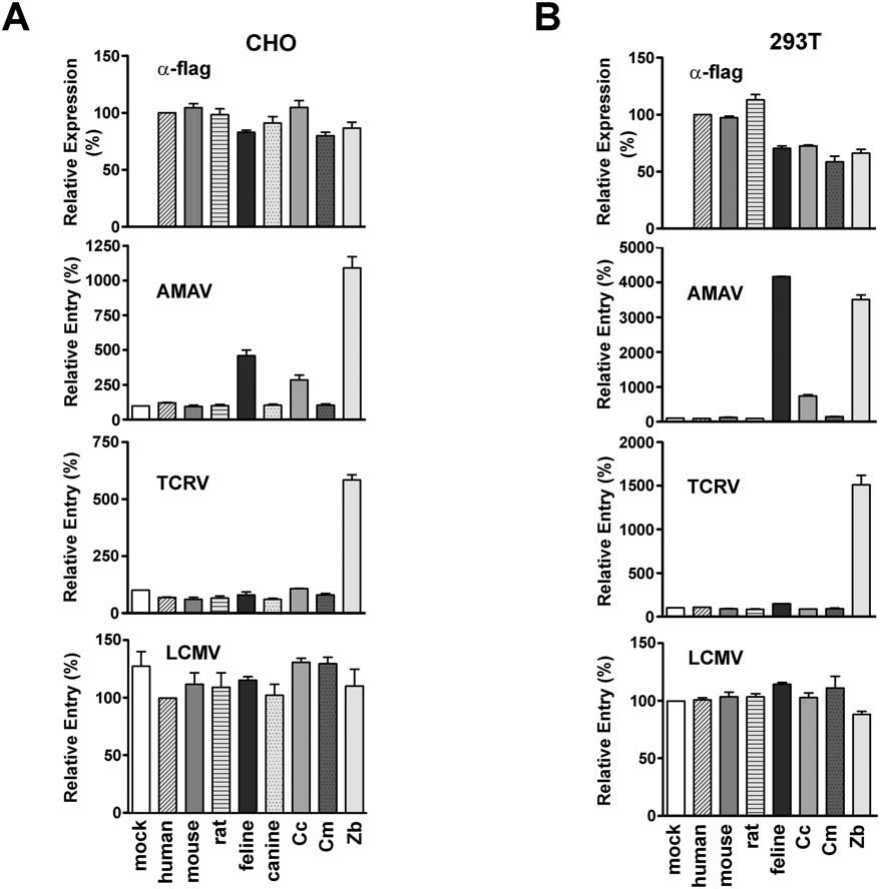
AMAV and TCRV pseudoviruses can use animal orthologs of TfR1. CHO cells ((A), left panel) were transfected with vector alone (mock) or plasmids encoding human, mouse, rat, feline, canine, *C. callosus* (Cc), *C. musculinus* (Cm), and *Z. brevicauda* (Zb) TfR1 orthologs. HEK293T cells ((B), right panel) were transfected with the same plasmids with the exception of the one encoding canine TfR1. Cell surface expression was determined 48 hr later by flow cytometry using an antibody directed against a flag tag present at the C-terminus of each ortholog. In parallel, aliquots of these cells were infected with AMAV, TCRV, or LCMV pseudoviruses. Infection levels were assessed as in Figure 2. Expression levels of various TfR1 were normalized to that of human TfR1 (α-flag, top panels). Infection levels were normalized to that of mock-transfected cells.

Phylogenetic analysis reveals that GTOV GP shares greater sequence similarity with AMAV GP than it does with the GPs of the other pathogenic NW arenaviruses (Figure 1). AMAV, however, does not use human TfR1 as a cellular receptor. We sought to determine if animal orthologs of TfR1 could support entry mediated by the GPs of AMAV and TCRV. Chinese hamster ovary (CHO) cells were transfected with plasmids expressing the human, mouse, rat, feline, canine, *Calomys callosus* (Cc ; MACV host), *Calomys musculinus* (Cm ; JUNV host), or *Zygodontomys brevicauda* (Zb ; GTOV host) orthologs of TfR1. TfR1 expression was assessed by flow cytometry using an antibody directed against a flag tag added to the C-terminus of each ortholog. In parallel, aliquots of the same cells were infected with AMAV or TCRV pseudoviruses. As these viruses readily enter CHO cells using a TfR1-independent pathway, pseudoviruses were diluted to minimize background entry. Pseudovirus bearing the GP of lymphocytic choriomeningitis virus (LCMV) was used as a control. As shown in Figure 3A, infection by AMAV and TCRV pseudoviruses was significantly enhanced by the introduction of ZbTfR1 into CHO cells, while AMAV pseudovirus entry was enhanced to a lesser degree by CcTfR1. The entry of AMAV pseudovirus was also enhanced by feline TfR1 expression. We and others have previously shown that this TfR1 ortholog is also used efficiently by the pathogenic viruses MACV, JUNV and GTOV [39],[40]. The entry of LCMV pseudovirus was unaffected. We repeated these experiments using HEK293T cells, and obtained similar results (Figure 3B). These data show that although AMAV and TCRV pseudoviruses do not use human TfR1, they nonetheless use the TfR1 orthologs of several mammalian species.

### AMAV and TCRV bind and use the TfR1 orthologs of their host animals

**Figure 4:**
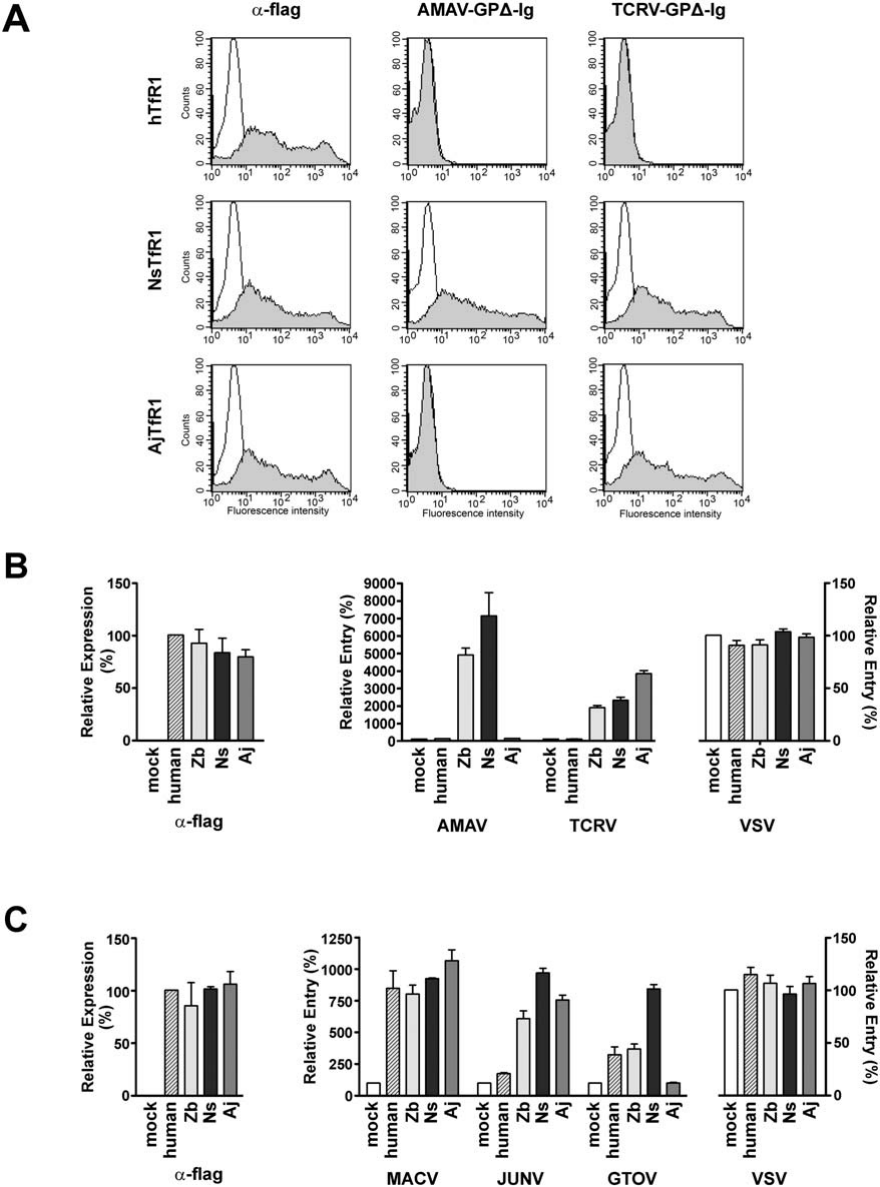
AMAV and TCRV GPs bind and use the TfR1 orthologs of their respective host species. (A) HEK293T cells were transfected with plasmids encoding human TfR1 (hTfR1), *N. spinosus* TfR1 (NsTfR1), or *A. jamaicensis* TfR1 (AjTfR1). Cells were incubated 48 hr later with an α-flag antibody or Ig-fusion proteins comprising the truncated GP1 subunits [32] of AMAV and TCRV (AMAV GP1Δ-Ig and TCRV GP1Δ-Ig, respectively). The association of these proteins with cells was measured by flow cytometry. The data shown are representative of two independent experiments, duplicated in each assay, with similar results. Mean fluorescence values for TfR1 ortholog expression were 435, 380, and 458 for human, Ns, and AjTfR1, respectively. Mean fluorescence values for AMAV GP1Δ-Ig binding to transfected cells were 1.5, 597, and 1.6, and those for TCRV GPΔ-Ig binding were 3.7, 425, and 553 for human, Ns, and AjTfR1, respectively. (B) HEK293T cells were transfected with plasmids encoding human, ZbTfR1, NsTfR1, AjTfR1 orthologs or vector alone. Cell surface expression of the TfR1 orthologs was determined as in Figure 3. Aliquots of these cells were infected with AMAV, TCRV, or VSV pseudoviruses, and infection levels were assessed as in Figure 2. (C) An experiment similar to the one performed in (B), except CHO cells were used for transfection and infected with MACV, JUNV, GTOV, or VSV pseudoviruses. Expression levels of the various TfR1 orthologs were normalized to that of human TfR1 (α-flag, left panels). Infection levels were normalized to that of mock-transfected cells.

We next investigated whether AMAV and TCRV GP could also use the TfR1 orthologs of their host species, the small rodent *N. spinosus* and the fruit bat *A. jamaicensis*, respectively. We cloned the genes of their TfR1 orthologs by RT-PCR from frozen tissues of *N. spinosus* (NsTfR1) and *A. jamaicensis* (AjTfR1). In order to determine the ability of the AMAV and TCRV GP1 proteins to bind these TfR1 orthologs, HEK293T cells were transfected with plasmids expressing human, Ns, or AjTfR1. These cells were incubated with Ig-fusion proteins comprising the GP1 subunits of AMAV or TCRV (AMAV-GP1Δ-Ig and TCRV-GP1Δ-Ig, respectively). The binding of these proteins to transfected cells was measured by flow cytometry using an α-human IgG antibody. The expression levels of TfR1 orthologs were assessed in parallel using an α-flag antibody. As shown in Figure 4A, while the expression levels of all TfR1 orthologs were comparable (left panels), AMAV-GP1Δ-Ig bound cells expressing NsTfR1, but did not bind cells expressing AjTfR1. TCRV-GP1Δ-Ig bound cells expressing AjTfR1 and also cells expressing NsTfR1. Neither Ig-fusion protein bound cells expressing human TfR1. Although both AMAV and TCRV pseudoviruses enter HEK293T cells using a TfR1-independent pathway, indicating the presence of an alternative receptor, we did not observe binding of AMAV or TCRV-GP1Δ-Ig to these cells (data not shown). This observation is consistent with an entry process dependent on a lower affinity receptor.

To determine if AMAV and TCRV pseudoviruses could use NsTfR1 and AjTfR1 to infect cells, HEK293T cells were transfected with plasmids expressing human TfR1, ZbTfR1, NsTfR1, AjTfR1, or vector alone, and then incubated with AMAV and TCRV pseudoviruses. Pseudovirus bearing the G protein of vesicular stomatitis virus (VSV) was used as a control. Consistent with the GP1Δ-Ig binding data, AMAV pseudovirus used only NsTfR1, while TCRV pseudovirus efficiently used both AjTfR1 and NsTfR1 (Figure 4B). We next assessed whether NsTfR1 and AjTfR1 could also serve as receptors for the pathogenic NW clade B arenaviruses. As the pathogenic NW arenaviruses efficiently enter HEK293T cells using endogenous human TfR1 [32], CHO cells were transfected with plasmids expressing human TfR1, AjTfR1, NsTfR1, ZbTfR1, or vector alone, and subsequently infected with MACV, JUNV, or GTOV pseudoviruses. As shown in Figure 4C, MACV and JUNV, like TCRV, used both NsTfR1 and AjTfR1, while GTOV, like the closely related AMAV, efficiently used NsTfR1 but not AjTfR1.

**Figure 5:**
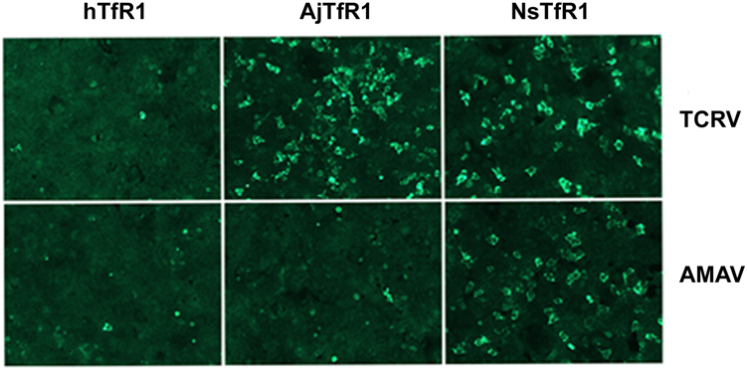
Host-animal orthologs of TfR1 support the entry of infectious AMAV and TCRV. HEK293 cells were transfected with plasmids encoding human TfR1, AjTfR1, or NsTfR1, and infectious viruses added at 36 hr post-transfection. Incubation was continued for 12 hr, and the cells were washed, fixed, and stained as described in Materials and Methods.

We confirmed these findings using replication-competent TCRV and AMAV viruses. HEK293 cells were transfected with plasmids expressing human TfR1, AjTfR1 or NsTfR1, and subsequently infected with replication-competent TCRV or AMAV. As shown in Figure 5, the infection of TCRV was enhanced on cells expressing AjTfR1 and NsTfR1, while that of AMAV was only enhanced on cells expressing NsTfR1.

### Modest changes convert human TfR1 into an efficient receptor for nonpathogenic NW arenaviruses

**Figure 6:**
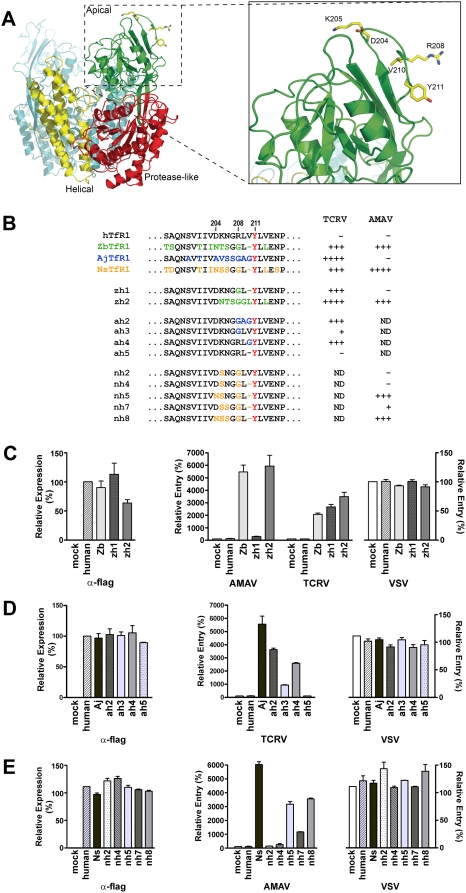
Modest mutations convert human TfR1 into an efficient receptor for AMAV and TCRV. (A) The structure of the human TfR1 dimer is shown, oriented with the cell membrane at the bottom. The apical, protease-like, and helical domains are indicated in green, red, and yellow, respectively, on one monomer. The other monomer is shown in cyan. In the right panel, the TfR1 apical domain is enlarged; a loop comprising residues 202-212, implicated as a site of interaction with the GPs of NW clade B arenaviruses, is shown. The side chains of residues D204, K205, R208, V210, and Y211 are colored yellow. The image was rendered using PyMol [59]. (B) Sequence alignment of residues 195 through 216 of human TfR1 with analogous sequences of the TfR1 orthologs of *Z. brevicauda* (ZbTfR1), *A. jamaicensis* (AjTfR1), and *N. spinosus* (NsTfR1). Variants of human TfR1 containing sequence from *Z. brevicauda* (zh1 and zh2), *A. jamaicensis* (ah2 through ah5), and *N. spinosus* (nh2, nh4, nh5, nh7, and nh8) TfR1 were generated based on this sequence alignment. Zb, Aj, and NsTfR1 sequences are shown in green, blue, and yellow, respectively. The right panel summarizes the entry data. ND = not determined. (C–E) HEK293T cells were transfected with plasmids encoding human, Zb, Aj, or NsTfR1 along with the zh variants (C), the ah variants (D), or the nh variants (E). The expression level of each TfR1 variant was assessed as in Figure 3. In parallel, cells were infected with AMAV, TCRV, or VSV pseudoviruses. The expression levels of the various TfR1 orthologs were normalized to that of human TfR1 (α-flag, left panels), and infection levels were normalized to that of mock-transfected cells.

A loop (residues 202–212) between β strands 1 and 2 in the apical domain of human TfR1 is a likely interaction site for the GPs of the NW hemorrhagic fever arenaviruses [39],[44]. This loop includes tyrosine 211, which is essential for MACV, JUNV, and GTOV to use human TfR1 [39]. We compared the sequences of ZbTfR1, NsTfR1, and AjTfR1 in this region with that of human TfR1, and found several differences between human and animal TfR1 (Figure 6A and 6B). We sought to determine whether replacing the human TfR1 residues with their equivalents from animal TfR1 orthologs would allow the modified human TfR1 to serve as a receptor for AMAV and TCRV pseudoviruses. Figure 6A shows a panel of eleven human TfR1 variants that were examined. In the first two variants (zh1 and zh2), residues from human TfR1 were replaced with those from ZbTfR1. Four variants (ah2 through ah5) include residues from AjTfR1, and five variants (nh2, nh4, nh5, nh7 and nh8) include NsTfR1 sequence. These variants were expressed in HEK293T cells, and the cells were incubated with TCRV or AMAV pseudoviruses. A variant (zh1) containing two alterations - R208G and ΔV210 - was an efficient receptor for TCRV (Figure 6C). As expected from comparison with zh1, a variant (ah2) containing three AjTfR1 residues in place of human TfR1 residues 208–210 (R208G, L209A, and V210G) was a functional receptor for TCRV (Figure 6D). When mutations at R208 and V210 were individually introduced into human TfR1, the variant containing V210G (ah4) supported TCRV pseudovirus entry efficiently, while the variant containing R208G (ah3) was a less efficient receptor. Deletion of V210 (ah5), however, did not convert human TfR1 into a receptor for TCRV. A slightly larger alteration in human TfR1 was necessary for AMAV pseudovirus infection; nh5, which contains four mutations - D204N, K205S, R208G, and ΔV210 - or nh8, which contains five mutations, were efficient receptors for AMAV (Figure 6E). The entry of VSV pseudovirus was unaffected by the introduction of any of the variants. Thus, only minimal alteration of human TfR1 was sufficient for it to serve as an efficient receptor for the nonpathogenic viruses AMAV and TCRV.

## Discussion

Five NW arenaviruses have been shown to cause hemorrhagic fever in humans. We have previously shown that four of these viruses – MACV, JUNV, GTOV, and SABV – require TfR1 to enter human cells [32]. We have also shown that these viruses utilize the TfR1 orthologs of their rodent hosts efficiently and with specificity [39]. A recently identified fifth hemorrhagic fever NW arenavirus, Chapare virus, remains to be characterized. Collectively, these data indicate a central role for TfR1 in the replication of NW clade B arenaviruses that cause hemorrhagic fever in humans.

Closely related to these pathogenic viruses are three clade B arenaviruses that do not cause disease in humans, namely TCRV, AMAV, and CPXV, although TCRV may have caused mild disease in a laboratory worker [10]. Here we have studied the two best characterized of these viruses, TCRV and AMAV. The GP sequences of these viruses are more similar to those of the pathogenic viruses than they are to each other (Figure 1). In spite of the similarity of AMAV and TCRV to the other members of their respective subclades, these nonpathogenic viruses do not utilize human TfR1. In contrast, every clade B virus shown to use human TfR1 causes a South American hemorrhagic fever [24], [32]–[35]. There is thus a close correlation between use of human TfR1 and the ability of a NW arenavirus to cause severe human disease. A possible exception is the Whitewater Arroyo virus (WWAV), a NW clade A/B arenavirus that does not appear to use human TfR1, but is tentatively associated with three human fatalities in 1999–2000 [45],[46]. Follow up studies by the CDC, however, failed to find any evidence of WWAV infection in the human tissues mentioned in the original article (Nichol, personal communication). In addition, there have been no further reports of WWAV infection being associated with human disease.

This correlation suggests that TfR1 use is a key determinant in the virulence of clade B viruses, which raises an important question: how easily might clade B viruses circulating in animals gain the ability to use human TfR1? Our findings shed light on this issue; TCRV and AMAV pseudoviruses efficiently utilize the TfR1 orthologs of several species including those of their native hosts, *A. jamaicensis* and *N. spinosus*, respectively (Figures 3 and 4). As with the pathogenic clade B viruses, AMAV and TCRV utilized most efficiently the TfR1 ortholog of their natural hosts. It is therefore likely that TfR1 is also central to the replication of these nonpathogenic viruses in their native hosts.

To investigate the possibility that gain-of-use of human TfR1 may not require extensive alteration of the TCRV and AMAV GPs, we sought to identify differences between human TfR1 and host animal TfR1 orthologs that determine the ability of these molecules to support infection by TCRV and AMAV pseudoviruses. We swapped residues in a loop in the apical domain of TfR1 that is a key determinant of host specificity (Figure 6). Surprisingly, alteration of a single residue (V210G, ah4) allowed human TfR1 to efficiently support the entry of TCRV pseudovirus, while replacement of four residues in this loop with the corresponding residues of NsTfR1 (nh5) was necessary to support efficient infection by AMAV pseudovirus (Figure 6D and 6E). Although these alterations are made to the receptor, it is reasonable to infer that similarly modest changes in the arenaviral GPs would also permit efficient use of human TfR1. For example, in order to bind human TfR1, TCRV GP would only need to acquire mutations that accommodate V210, present in human TfR1 but absent or replaced by a glycine in animal TfR1 orthologs. Our data, although limited, suggest that small alterations in the arenaviral GP could convert these nonpathogenic NW arenaviruses into human pathogens.

This possibility has precedent in other emerging viruses. For example, alteration of only a few residues in the spike protein of the severe acute respiratory syndrome coronavirus (SARS-CoV) was sufficient for this glycoprotein, derived from an animal isolate of the virus, to efficiently use the human ortholog of its receptor, angiotensin converting enzyme 2 (ACE2) [47]. Similarly, the adaptation of avian influenza viruses to humans is thought to require as few as two amino acid substitutions in the influenza hemagglutinin glycoprotein (HA). These mutations allow HA to bind its human receptor, α2,6 -linked sialosides [48],[49]. Structural studies of the SARS-CoV and influenza A virus entry proteins in complex with their respective receptors have helped to identify and predict entry protein alterations that contribute to the ability of these viruses to cause human disease [48],[50]. Similar studies of arenanaviral GP molecules bound to TfR1 are likely to further elucidate the risk posed by AMAV, TCRV, and perhaps other nonpathogenic arenaviruses.

Of note in this regard, bats are increasingly recognized as reservoirs for viruses that can cross species barriers to infect humans [51]. Nipah, Hendra, and perhaps SARS-CoV and Ebola viruses, are on the list of emerging viruses that involve amplification in bat hosts [52]. Although TCRV was isolated from bats [19], the suggestion that Artibeus species bats serve as the natural hosts for TCRV has been questioned [53]. Our observation that AjTfR1 is an efficient receptor for TCRV, and also for JUNV and MACV, supports a role for bats in sustaining the replication of arenaviruses. Ecological surveys of bats for novel arenaviruses are thus warranted, and may afford further insight into the diversity of arenaviruses. In addition, although cats experimentally infected with MACV do not become ill [54], viral titers in these animals were not determined. The finding that feline TfR1 supports the entry of AMAV and the pathogenic clade B arenaviruses thus highlights a possible role for cats in serving as intermediate hosts in the transmission of NW arenaviruses to humans [39],[40].

Despite the inability of AMAV and TCRV to cause disease in humans, their GP proteins mediate entry into human cells through a pathway independent of human TfR1 or α-dystroglycan (Figure 2) [40],[43]. This observation suggests that these viruses can use more than one receptor, and raises the possibility that the pathogenic clade B arenaviruses may also use an additional receptor [40]. Indeed, certain strains of the Old World arenavirus lymphocytic choriomeningitis virus (LCMV) use both α-dystroglycan dependent and independent pathways to infect cells [55]. An alternative receptor may substitute for TfR1, or function downstream of TfR1 in a manner analogous to the HIV-1 coreceptors. Identification of such a receptor on human cells will likely broaden our understanding of the entry mechanisms of a number of NW arenaviruses.

## Materials and Methods

### Phylogeny

ClustalW was used to align the GP sequences of the NW arenaviruses and for phylogenetic calculations, and the cladogram was plotted using Drawtree [56],[57]. The sequences used in the analysis are those of full-length GPs: ALLV, Allpahuayo virus (strain CHLP 2472, GenBank accession no. AY012687); AMAV, Amapari virus (BeAn 70563, AF512834); BCNV, Bear Canyon virus (A0060209, AF512833); Catarina virus (AV A0400212, DQ865245); Chapare virus (810419, EU260463); CPXV, Cupixi virus (BeAn 119303, AF512832); FLEV, Flexal virus (BeAn HEK293922, AF512831); GTOV, Guanarito virus (strain INH 95551, NC_005077); JUNV, Junín virus (MC2, D10072); LATV, Latino virus (MARU 10924, AF512830); MACV, Machupo virus (Carvallo, NC_005078); OLVV, Oliveros virus (3229 1, U34248); PARV, Paraná virus (12056, AF512829); PICV, Pichindé virus (AN3739, NC_006447); PIRV, Pirital virus (VAV 488, AF277659); SABV, Sabiá virus (SPH114202, NC_006317); Skinner Tank virus (AV D1000090, EU123328); TCRV, Tacaribe virus (TRVL 11573, NC_004HEK293); TAMV, Tamiami virus (W 10777, AF512828); WWAV, Whitewater Arroyo virus (AV 9310135, AF228063).

### Cells and plasmids

HEK293T cells (human embryonic kidney, ATCC, CRL-11268) were maintained in Dulbecco's modified Eagle's medium, and CHO cells (Chinese hamster ovary epithelial, ATCC, CCL-61) in F12 medium. Both cell lines were grown in the presence of 10% fetal bovine serum. Plasmids encoding human, mouse, rat, *C. callosus* (large vesper mouse), and *C. musculinus* (drylands vesper mouse), *Z. brevicauda* (short-tailed cane mouse) TfR1 are previously described [39]. The plasmids expressing the TfR1 orthologs of *F. domesticus* (cat) and *C. familiaris* (dog) were generously provided by Colin Parrish (Cornell University, Ithaca, NY). TfR1 genes for *Neacomys spinosus* and *Artibeus jamaicensis* were cloned from frozen heart and kidney, and liver tissues, respectively. Deposited sequences in GenBank (FJ154604 and FJ154605) cross-reference to catalog numbers of the Museum of Southwestern Biology, University of New Mexico from where these specimens were obtained. RNA was isolated from these tissues using RNAqueous (Ambion), and cDNA was generated by SuperScript III First-Strand Synthesis system for RT-PCR (Invitrogen). PCR primers used to amplify the genes for both NsTfR1 or AjTfR1 orthologs are: sense 5′ ATGATGGATCAAGCCAGATCAGCAWTCTCT 3′ and anti-sense 5′ AAAYTCATTGTCAATGTCCCAAAYGTCACCA 3′. These genes, fused with a C-terminal flag tag, were cloned into the pcDNA3.1 expression vector (Invitrogen). Amino acid substitutions in human TfR1 were performed using the QuikChange method for site-directed mutagenesis (Stratagene).

AMAV GP1Δ-Ig and TCRV GP1Δ-Ig were generated by PCR-amplification of AMAV GP1 residues 80–228 and TCRV GP1 residues 87–243, with boundary residues selected according to a similar construct of MACV GP1 previously shown to bind most efficiently to Vero cells [32]. These fragments were cloned into a previously described pcDM8-based plasmid containing the CD5 signal sequence and the Fc region of human IgG1 [58]. MACV (Carvallo strain), JUNV (MC2), and GTOV (INH-95551), GP-expressor plasmids have been described previously [32]. Genes for AMAV and TCRV GP are gifts from Stefan Kunz (University of Lausanne, Switzerland); they were PCR amplified from infected Vero cells, and cloned into pCAGGS expression vector. The sequence of AMAV GP is the same as BeAn 7063 strain (GenBank AF512834), and that of TCRV has a deletion spanning amino acid residues 121–132, and substitutions at three residues, I134A, G418S and E458R, compared to GenBank NC_004293.

### GP1Δ-Ig binding assays

AMAV GP1Δ-Ig and TCRV GP1Δ-Ig binding to various TfR1 orthologs was detected by flow cytometry. Ig-fusion proteins were produced in Free Style HEK293 Expression Medium (Invitrogen) from HEK293T cells transfected with the appropriate plasmid, purified by binding to protein-A Sepharose and eluted with 3 M MgCl_2_, dialyzed in PBS, and concentrated using a YM50 filter unit (Millipore). For binding assays, HEK293T cells were transfected with the various TfR1-encoding plasmids. After 48 hr, cells were detached by 1 mM EDTA/PBS and incubated in 100 ul of PBS-2% goat serum containing 0.5 ug of either an α-flag M2 monoclonal antibody (Sigma) or the indicated Ig-fusion protein. Cells were then stained with α-mouse IgG, or with α-human IgG (Fc-specific) antibodies conjugated with phycoerythrin (Jackson Immuno Laboratories), respectively.

### Pseudovirus infection

To generate recombinant retroviruses pseudotyped with arenaviral GP molecules, HEK293T cells were transfected at a 1∶1∶1 ratio with plasmids encoding the respective arenaviral GP, the MLV *gag* and *pol* genes, and the pQCXIX retroviral vector (BD Biosciences) expressing GFP, as previously described [32]. Cell supernatants were harvested 24 hr post-transfection, and filtered through a 0.45 µm filter. HEK293T cells or CHO cells were transfected with plasmids expressing various TfR1 orthologs, and at 24 hr, the cells were replated for flow cytometry and infection. At 48 hr post-transfection, TfR1 expression levels were assessed by flow cytometry using an α-flag M2 antibody. In parallel, cells were infected with the indicated pseudoviruses for 2 hours. Twenty-four (HEK293T cells) or forty-eight (CHO cells) hours after infection, the cells were detached with trypsin and the GFP expression levels were measured by flow cytometry, and normalized to that of mock-transfected cells. Values are the average of 2–5 duplicated experiments.

### Infection of live arenaviruses

Infection of HEK293 cells with replication-competent TCRV (TRVL11573) or AMAV (BeAn70563), was performed in the BSL3 laboratory at the Special Pathogens Branch, Centers for Disease Control and Prevention, Atlanta. Infections and immunofluorescence staining were performed as described previously [32]. Briefly, HEK293 cells growing on gelatin-treated glass coverslips were transfected with plasmids encoding AjTfR1, NsTfR1, or human TfR1. Thirty-six hours later, these cells were infected with TCRV or AMAV at a multiplicity of infection of 2.0. At 12 hr post-infection, the cells were washed, fixed with 3% formaldehyde, permeabilized with 0.1% Triton X-100 and incubated with mouse hyperimmune sera specific for NW arenaviruses. Cells were then washed and incubated with a FITC-conjugated secondary antibody.
